# Some Current in Diet

**Published:** 1901-04-06

**Authors:** 


### SOME CURRENT ABSURDITIES IN DIET.

In an address on the subject of chronic Bright's disease recently delivered before the Halifax Medical Society, Dr. Alfred Barrs pointed out how absurd were some of the restrictions as to diet which are sometimes imposed upon patients suffering from this disorder. In laying down a diet for such a case one must remember, he said, that chronic Bright's disease is a very chronic disorder, and when properly managed is not incompatible with much usefulness and not a little enjoyment of life, and we ought to take care that such usefulness and enjoyment are not unnecessarily curtailed by anything we may do. We do not keep all patients suffering from this disease in bed to the end of their days, neither ought we to keep them continuously on restricted diets.

To limit the input of proteid material so as to bring the work of the kidney within its capacity is to act on scientific lines; but we know so little of the more remote stages of nitrogenous metabolism that beyond this very general statement we cannot safely go. Yet there is no disease in which the tyranny of diet has been so recklessly practised. To live for weeks and weeks on a purely milk diet is not necessary, is indeed distinctly harmful; and there is no clear evidence that it meets the scientific indication. It has been shown that the output of nitrogen is greater on a purely milk diet than on an ordinary mixed diet, so that as far as the urea output is concerned, there is no theoretical advantage in such a diet, and clinical experience in chronic Bright's disease shows the same thing. Dr. Barr's rule is simplicity itself. He says that if the bowels are acting freely the patient may live on such ordinary mixed diet, including meat, as he has an appetite for and can digest. Patients who are confined to bed and suffering from uræmic vomiting or diarrhœa, and are getting towards the end of the disease, cannot of course have any appetite, and the difficulty is to contrive food of any kind for them. The distinctions, again, which we draw between different kinds of meat in dieting cases of renal disease and gout are ridiculous. A patient may eat chicken and fish, and not mutton, mutton and not beef, and so on. There is no reason for this. Pork, which is white, and in the case of Alexis St. Martin was shown to be the most digestible of meats, is usually entirely forbidden. Van Noorden mentions the case of a patient with chronic parenchymatous nephritis who took half a pound of poultry daily for five days, during which he excreted the same amount of nitrogen, and a trifle more albumen than he did in the next five days, in which instead of poultry he took an equivalent amount of nitrogen in beef. Water and other fluid sustenance should be taken freely, but alcohol is perhaps as well avoided if cardiac failure does not require it.

